# Supplementary material for: Buffering of genetic defects in animal development by regeneration programs
Source: bioRxiv. 2025 Oct 26:2025.10.25.684558. Preprint. [Version 1] doi: 10.1101/2025.10.25.684558 (PMC12633463; doi:10.1101/2025.10.25.684558)
Supplement: 1 [file NIHPP2025.10.25.684558V1-supplement-1.pdf]

bioRxiv preprint doi: https://doi.org/10.1101/2025.10.25.684558; this version posted October 26, 2025. The copyright holder for this preprint (which was not certified by peer review) is the author/funder, who has granted bioRxiv a license to display the preprint in perpetuity. It is made available under aCC-BY 4.0 International license.

Supplementary Information

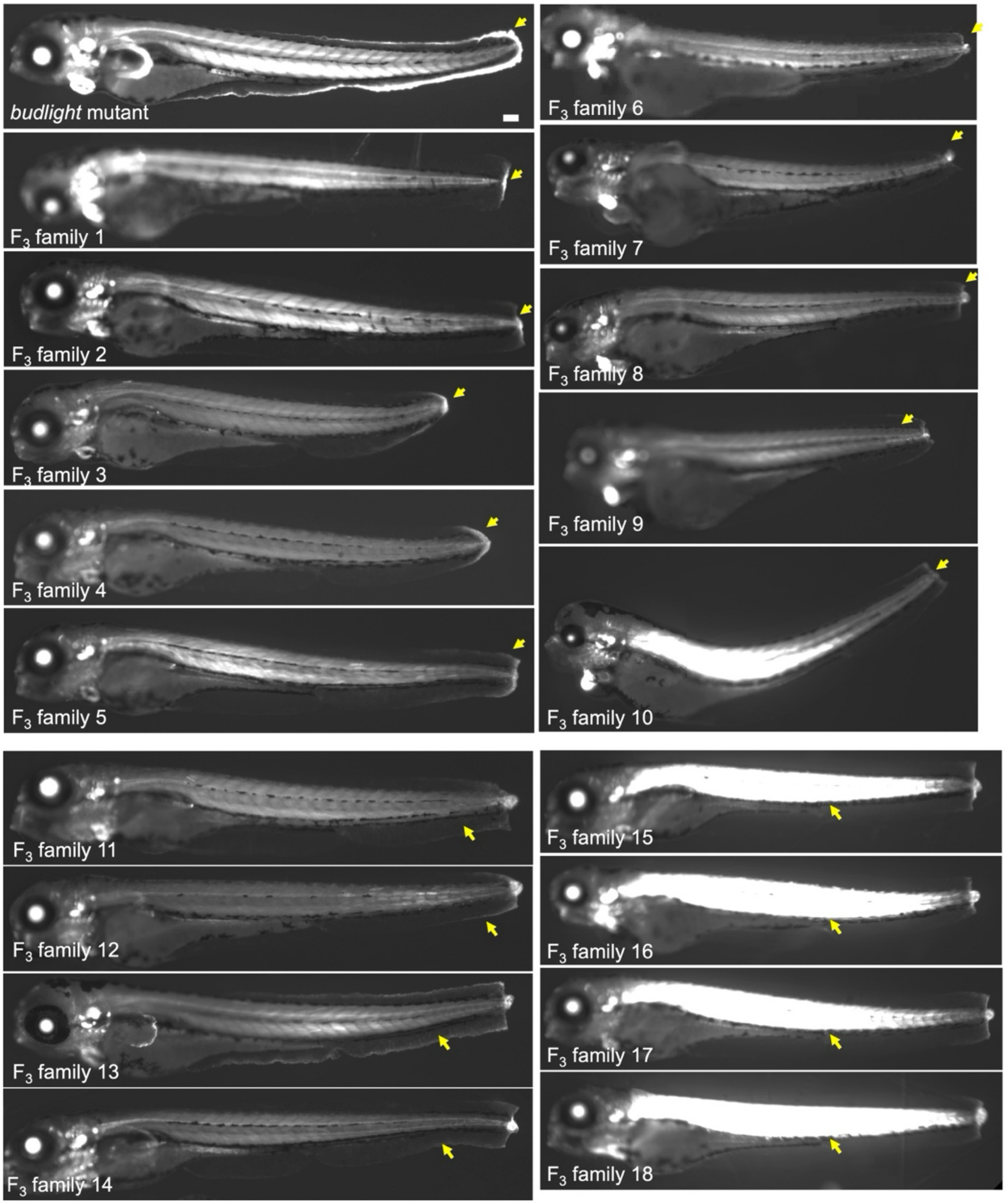

Fig S1. Phenotypes of F_3 larvae. Ten F_3 families contain larvae with limited regeneration phenotypes (yellow arrows) associated with EGFP expression, in both 1st and 2nd repeated screenings. F_3 family 1-5 displayed reduced size of regenerating tissues, and F_3 family 5-9 displayed EGFP expression at the edge of finfold. One of the mutants (named "*budlight*") exhibited a strong regeneration defect linked to severely degenerated finfold in the absence of injury. Scale bar is 100 μm.

bioRxiv preprint doi: https://doi.org/10.1101/2025.10.25.684558; this version posted October 26, 2025. The copyright holder for this preprint (which was not certified by peer review) is the author/funder, who has granted bioRxiv a license to display the preprint in perpetuity. It is made available under aCC-BY 4.0 International license.

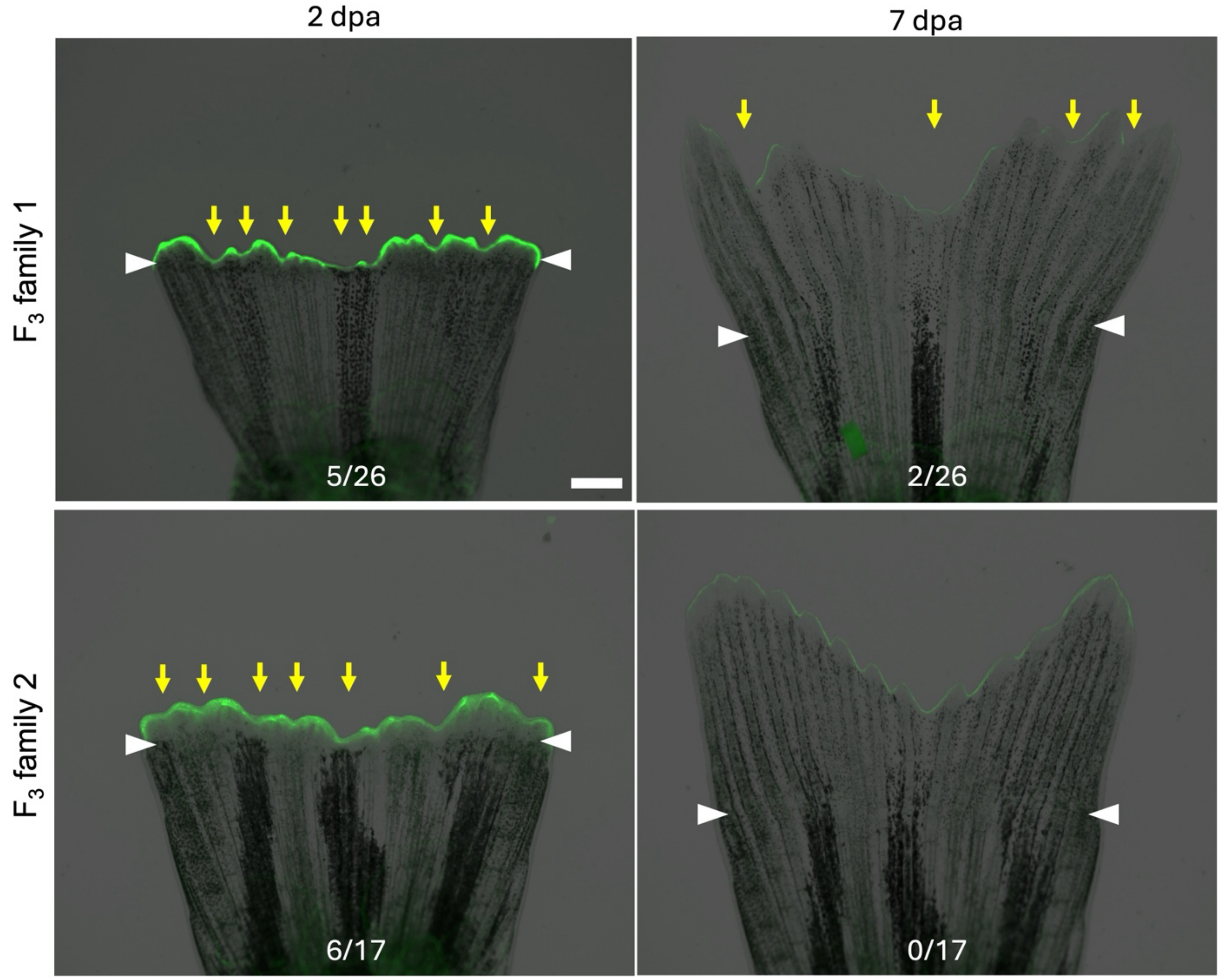

Fig S2. F_3 adults with limited regenerating tissues. Two families exhibiting a regeneration phenotype in Fig. S1A contain adult fish with abnormal regeneration (Yellow arrows) at 2 dpa during caudal fin regeneration (left panels), although fin regeneration was grossly normal at 7 dpa (right panels). Ratios representing abnormal regeneration among total family members are at bottom of images. White arrow heads indicate amputation planes. Scale bar is 1 mm.

bioRxiv preprint doi: https://doi.org/10.1101/2025.10.25.684558; this version posted October 26, 2025. The copyright holder for this preprint (which was not certified by peer review) is the author/funder, who has granted bioRxiv a license to display the preprint in perpetuity. It is made available under aCC-BY 4.0 International license.

SI Appendix, Table S1.

Table S1 lists the guide RNA targeting sequences (Length, 20 nt) used for the F_0 screen of zebrafish orthologues of human congenital defect associated genes. For all genes, a combination of 2-3 guide RNAs in equimolar quantities was used.
